# Supplementary material for: Investigating the diverse potential of a multi-purpose legume, Lablab purpureus (L.) Sweet, for smallholder production in East Africa
Source: PLoS One. 2020 Jan 27;15(1):e0227739. doi: 10.1371/journal.pone.0227739 (PMC6984688; doi:10.1371/journal.pone.0227739)
Supplement: S1 Table — Numbers in parentheses standard errors. (DOCX) [file pone.0227739.s001.docx]

**S1 Table.** **Soil properties of study site.** Numbers in parentheses standard errors.

|  |  | Block 1 | | | | | Block 2 | | | | | Block 3 | | | | |
| --- | --- | --- | --- | --- | --- | --- | --- | --- | --- | --- | --- | --- | --- | --- | --- | --- |
| Location | Depth | pH | EC  (uS/cm) | P  (mg/kg) | Sand % | Clay % | pH | EC  (uS/cm) | P (mg/kg) | Sand  % | Clay % | pH | EC  (uS/cm) | P  (mg/kg) | Sand  % | Clay  % |
| SARI | 0-20 cm | 6.79  (0.18) | 237.47 (45.3) | 9.78  (0.29) | 40% (2.35) | 40% (2.38) | 6.66 (0.06) | 167.37 (5.19) | 9.38 (0.75) | 37% (1.15) | 43% (1.33) | 6.69 (0.06) | 155.57 (5.82) | 4.96 (0.75) | 39% (1.15) | 43% (0.67) |
|  | 20-40 cm | 7.00 (0.07) | 138.57 (16.7) | 15.88 (2.71) | 37% (1.07) | 42% (2.03) | 6.92 (0.03) | 135.23 (5.75) | 21.42 (0.36) | 37% (1.76) | 45% (1.20) | 6.87 (0.07) | 146.23 (8.68) | 21.17 (6.87) | 38% (0.33) | 45% (0.33) |
