# Supplementary material for: Investigating the diverse potential of a multi-purpose legume, Lablab purpureus (L.) Sweet, for smallholder production in East Africa
Source: PLoS One. 2020 Jan 27;15(1):e0227739. doi: 10.1371/journal.pone.0227739 (PMC6984688; doi:10.1371/journal.pone.0227739)
Supplement: S2 Table — (DOCX) [file pone.0227739.s002.docx]

| **S2. Table. Type 3 ANOVA of lablab grain yield, biomass, and maize yield** | | | | |
| --- | --- | --- | --- | --- |
| Effect |  | df | F | p |
| Grain yield | Environment | (3,8) | 21 | 0.0004 |
| (kg ha^-1^) | Accession | (13,207) | 9.72 | <.0001 |
|  | Env x Accession | (39,207) | 5.62 | <.0001 |
|  | Maize Intercrop | (1,8) | 3.28 | 0.1077 |
|  | Env x Intercrop | (3,8) | 1.15 | 0.3849 |
|  | Intercrop x Accession | (13,207) | 0.35 | 0.9831 |
|  | Env x Acc x Int | (39,207) | 0.51 | 0.9926 |
| Biomass | Environment | (3,8) | 50.8 | <.0001 |
| (Mg ha^-1^) | Accession | (13,207) | 3.42 | <.0001 |
|  | Env x Accession | (39,207) | 1.97 | 0.0013 |
|  | Maize Intercrop | (1,8) | 92.8 | <.0001 |
|  | Env x Intercrop | (3,8) | 19.5 | 0.0005 |
|  | Intercrop x Accession | (13,207) | 1.02 | 0.4306 |
|  | Env x Acc x Int | (39,207) | 0.98 | 0.5125 |
| Maize yield | Environment | (3,8) | 10.6 | 0.0036 |
| (kg ha^-1^) | Accession | (13,98) | 1.06 | 0.3996 |
|  | Env x Accession | (39,98) | 0.93 | 0.5918 |
