# Supplementary material for: Investigating the diverse potential of a multi-purpose legume, Lablab purpureus (L.) Sweet, for smallholder production in East Africa
Source: PLoS One. 2020 Jan 27;15(1):e0227739. doi: 10.1371/journal.pone.0227739 (PMC6984688; doi:10.1371/journal.pone.0227739)
Supplement: S3 Table — (DOCX) [file pone.0227739.s003.docx]

| **S3 Table.** LER for lablab | | |
| --- | --- | --- |
|  | Grain LER | Biomass LER |
| SARI 2016 | 3.21 | 2.56 |
| SARI 2017 | 2.37 | 2.10 |
| TPRI 2016 | 2.82 | 1.76 |
| TPRI 2017 | -- | 1.59 |
