# Supplementary material for: Investigating the diverse potential of a multi-purpose legume, Lablab purpureus (L.) Sweet, for smallholder production in East Africa
Source: PLoS One. 2020 Jan 27;15(1):e0227739. doi: 10.1371/journal.pone.0227739 (PMC6984688; doi:10.1371/journal.pone.0227739)
Supplement: S4 Table — Measurements from sole cropped plots only. (DOCX) [file pone.0227739.s004.docx]

| **S4 Table. Correlation matrix from PCA of SARI 2017 data.** Measurements from sole cropped plots only. | | | | | | | | |
| --- | --- | --- | --- | --- | --- | --- | --- | --- |
| Correlation Matrix | | | | | | | | |
|  | Grain Yield | Soil Nitrate | Biomass | Nodule weight | δ^15^N | Maturity | Plant pop | %N |
| Grain yield | **1.0000** | 0.0330 | 0.3564 | 0.3800 | 0.0182 | -.4212 | 0.2977 | -.4633 |
| Soil Nitrate | 0.0330 | **1.0000** | 0.0031 | 0.0848 | -.0518 | 0.0677 | 0.2133 | -.1937 |
| Biomass | 0.3564 | 0.0031 | **1.0000** | 0.1382 | 0.2230 | -.4946 | 0.3939 | -.5304 |
| Nodule weight | 0.3800 | 0.0848 | 0.1382 | **1.0000** | -.1802 | 0.2290 | -.0477 | -.2146 |
| δ^15^N | 0.0182 | -.0518 | 0.2230 | -.1802 | **1.0000** | -.2311 | -.2572 | -.0600 |
| Maturity | -.4212 | 0.0677 | -.4946 | 0.2290 | -.2311 | **1.0000** | -.4409 | 0.2376 |
| Plant pop | 0.2977 | 0.2133 | 0.3939 | -.0477 | -.2572 | -.4409 | **1.0000** | -.4472 |
| % N | -.4633 | -.1937 | -.5304 | -.2146 | -.0600 | 0.2376 | -.4472 | **1.0000** |
