# Supplementary material for: Investigating the diverse potential of a multi-purpose legume, Lablab purpureus (L.) Sweet, for smallholder production in East Africa
Source: PLoS One. 2020 Jan 27;15(1):e0227739. doi: 10.1371/journal.pone.0227739 (PMC6984688; doi:10.1371/journal.pone.0227739)
Supplement: S5 Table — (DOCX) [file pone.0227739.s005.docx]

| **S5. Table. Type 3 ANOVA of PC1 and PC2 from PCA of SARI 2017 data** | | | | |
| --- | --- | --- | --- | --- |
| Effect |  | df | F | p |
| PC1 | Accession | (13,21) | 3.70 | 0.0038 |
|  | Block | (2,21) | 8.53 | 0.0019 |
| PC2 | Accession | (13,21) | 0.88 | 0.5833 |
|  | Block | (2,21) | 2.54 | 0.1030 |
