# Supplementary material for: Investigating the diverse potential of a multi-purpose legume, Lablab purpureus (L.) Sweet, for smallholder production in East Africa
Source: PLoS One. 2020 Jan 27;15(1):e0227739. doi: 10.1371/journal.pone.0227739 (PMC6984688; doi:10.1371/journal.pone.0227739)
Supplement: S6 Table — Nodules sampled in sole cropped plots only. (DOCX) [file pone.0227739.s006.docx]

**S6 Table. Characterization of nodules sampled at SARI 2017 site.** Nodules sampled in sole cropped plots only.

| Accession Name | Accession Number | Nodules per Plant (#) | Nodule weight per plant (g) | Pink Colored Nodules (%) |
| --- | --- | --- | --- | --- |
| CIAT 22759 | 1 | 9.6 | 0.26 | 88 |
| DL1001 | 3 | 4.6 | 0.15 | 75 |
| DL1002 | 4 | 8.5 | 0.19 | 58 |
| Echo Cream | 6 | 8.8 | 0.16 | 85 |
| Highworth | 8 | 2.0 | 0.07 | 66 |
| ILRI 13700 | 12 | 13.0 | 0.18 | 79 |
| ILRI 14437 | 14 | 9.6 | 0.20 | 83 |
| ILRI 6930 | 16 | 8.4 | 0.11 | 81 |
| Karamoja Red | 17 | 7.1 | 0.11 | 84 |
| PI 195851 | 21 | 7.6 | 0.19 | 84 |
| Q 6880B | 22 | 4.5 | 0.09 | 52 |
| Rongai | 23 | 7.3 | 0.16 | 77 |
| SARI Nyeupe | 25 | 11.4 | 0.18 | 82 |
| SARI Rongai | 26 | 11.6 | 0.25 | 74 |
