# Supplementary material for: Investigating the diverse potential of a multi-purpose legume, Lablab purpureus (L.) Sweet, for smallholder production in East Africa
Source: PLoS One. 2020 Jan 27;15(1):e0227739. doi: 10.1371/journal.pone.0227739 (PMC6984688; doi:10.1371/journal.pone.0227739)
Supplement: S7 Table — (DOCX) [file pone.0227739.s007.docx]

| **S7 Table. Soil nitrate from 0-20 cm depth** | | |
| --- | --- | --- |
| Accession | SARI 2016  (ug/g) | SARI 2017  (ug/g) |
| 1 | 0.99 | 1.35 |
| 3 | 1.12 | 1.12 |
| 4 | 1.24 | 0.91 |
| 6 | 1.19 | 1.03 |
| 8 | 1.05 | 1.04 |
| 12 | 1.05 | 1.31 |
| 14 | 1.03 | 1.00 |
| 16 | 1.00 | 1.05 |
| 17 | 1.03 | 1.03 |
| 21 | 1.34 | 1.00 |
| 22 | 1.00 | 1.28 |
| 23 | 0.98 | 1.00 |
| 25 | 1.12 | 1.05 |
| 26 | 0.98 | 1.17 |
| Lablab Avg | 1.08 | 1.10 |
| Cowpea Avg | 1.33 | 1.35 |
