# Supplementary material for: Investigating the diverse potential of a multi-purpose legume, Lablab purpureus (L.) Sweet, for smallholder production in East Africa
Source: PLoS One. 2020 Jan 27;15(1):e0227739. doi: 10.1371/journal.pone.0227739 (PMC6984688; doi:10.1371/journal.pone.0227739)
Supplement: Attachment — Submitted filename: Response_to_Reviewers_PlosOne.docx [file pone.0227739.s009.docx]

Title: “Investigating the diverse potential of a multi-purpose legume, Lablab purpureus (L.) Sweet, for smallholder production in East Africa”

PONE-D-19-27128

Dear PLOS ONE Editor,

Below are our responses to the comments provided by the reviewer and academic editor.

We have revised the submission to address all comments, editor and reviewer. In response to the journal requirements highlighted by the academic editor, we have edited the manuscript to comply with PLOS ONE’s style requirements. Concerning Figure 6 which was highlighted by the editor as possibly being copyright material, we have clarified in the caption that we produced this map, so it is not copyrighted. We added information for clarification in the figure heading stating that the map was produced by us using QGIS software and public domain data, which does not impose any restrictions on maps produced and therefore is not copyrighted. Additionally, captions for supporting information files have been added at the end of the manuscript as requested (on page 35).

We trust these revisions address the edits suggested by the editor and reviewer.

Sincerely,

Alison Nord

Response to Reviewer #1:

The duration of legumes impact their tolerance to abiotic stresses as selected cultivars are with early to very late duration. Hence for assessing their suitability one has to compare similar duration cultivars.

**Response: We appreciate this point by the reviewer but would like to clarify that within this study we are looking at variation both across and within growth types, and therefore used the comprehensive statistical model shown on page 10 to assess cultivar performance.**

Both edaphic and weather conditions varied in selected two sites of experimentation. The information on one site soil conditions was provided. A comparison need to be done on impact of soil conditions while assessing the performance of cultivars.

**Response: We have provided the soil conditions as supplementary information as soil conditions of our study sites were not an explicit factor in the analysis (as only two sites were represented).**

Too many parameters were included for identifying suitable cultivar/s for biomass and seed yield. Nitrogen component needs to be addressed separately and some more detailed experimentation is required, hence this parameter may be omitted.

**Response: We have added clarification in the methods section on page 9 to highlight that the PCA performed for one site year was part of data collected as a sub study on nitrogen and productivity. This specific analysis looked at variability across accessions for parameters related to nitrogen fixation and productivity, not the overall suitability of cultivars for biomass and grain yield which was addressed in the larger study. As such, we feel it is important to include this sub study as an initial step towards understanding N dynamics across lablab cultivars for which research is currently lacking in the literature.**

Many statements are repetitive and lengthy such as explanation of results for two sites for two years may be made as single statement

**Response: We have addressed this concern and have made efforts throughout the manuscript to condense statements, as seen through the manuscript track changes. See especially page 14 of the results.**
